# Symptoms and probabilistic anatomical mapping of lacunar infarcts

**DOI:** 10.1186/s42466-020-00068-y

**Published:** 2020-08-03

**Authors:** Ewgenia Barow, Hans Pinnschmidt, Florent Boutitie, Alina Königsberg, Martin Ebinger, Matthias Endres, Jochen B. Fiebach, Jens Fiehler, Vincent Thijs, Robin Lemmens, Keith W. Muir, Norbert Nighoghossian, Salvador Pedraza, Claus Z. Simonsen, Christian Gerloff, Götz Thomalla, Bastian Cheng, on behalf of the WAKE-UP investigators

**Affiliations:** 1 Klinik und Poliklinik für Neurologie, Kopf- und Neurozentrum, University Medical Center Hamburg-Eppendorf, Martinistr. 52, 20246 Hamburg, Germany; 2 Institut für Medizinische Biometrie und Epidemiologie, University Medical Center Hamburg-Eppendorf, Martinistr. 52, 20246 Hamburg, Germany; 3 Hospices Civils de Lyon, Service de Biostatistique, F-69003 Lyon, France; 4 Klinik für Neurologie, Medical Park Berlin Humboldtmühle, An der Mühle 2-9, 13507 Berlin, Germany; 5 Centrum für Schlaganfallforschung Berlin (CSB), Charité - Universitätsmedizin Berlin, Charitéplatz 1, 10117 Berlin, Germany; 6 Klinik und Hochschulambulanz für Neurologie, Charité-Universitätsmedizin Berlin, Charitéplatz 1, 10117 Berlin, Germany; 7 Department of Diagnostic and Interventional Neuroradiology, University Medical Center Hamburg-Eppendorf, Martinistr. 52, 20246 Hamburg, Germany; 8 Stroke Division, Florey Institute of Neuroscience and Mental Health, University of Melbourne, 245 Burgundy Street, Heidelberg, VIC 3084 Australia; 9 Austin Health, Department of Neurology, 145 Studley Road, Heidelberg, VIC 3084 Australia; 10 Department of Neurology, University Hospitals Leuven, Herestraat 49, 3000 Leuven, Belgium; 11 KU Leuven – University of Leuven, Department of Neurosciences, Experimental Neurology, Oude Markt 13, 3000 Leuven, Belgium; 12 VIB, Center for Brain & Disease Research, Laboratory of Neurobiology, Campus Gasthuisberg, Herestraat 49, 3000 Leuven, Belgium; 13 Institute of Neuroscience & Psychology, University of Glasgow, University Avenue, Glasgow, G12 8QQ UK; 14 Department of Stroke Medicine, Hospices Civils de Lyon, Université Claude Bernard Lyon 1, Lyon, France; 15 Department of Radiology, Institut de Diagnostic per la Image (IDI), Hospital Dr Josep Trueta, Institut d’Investigació Biomèdica de Girona (IDIBGI), Parc Hospitalari Martí i Julià de Salt - Edifici M2, 17190 Salt, Girona, Italy; 16 Department of Neurology, Aarhus University Hospital, 8200 Aarhus, Denmark

**Keywords:** Lacunar infarct, Magnetic resonance imaging, Lesion distribution, Probabilistic atlas, WAKE-UP

## Abstract

**Background:**

The anatomical distribution of acute lacunar infarcts has mainly been studied for supratentorial lesions. In addition, little is known about the association with distinct stroke symptoms, not summarized as classical lacunar syndromes. We aimed to describe the spatial lesion distribution of acute supra- and infratentorial lacunar infarcts and their association with stroke symptoms in patients eligible for thrombolysis.

**Methods:**

All patients enrolled in the WAKE-UP trial (efficacy and safety of magnetic resonance imaging [MRI]-based thrombolysis in wake-up stroke) were screened for lacunar infarcts on diffusion-weighted imaging (DWI). The relationship between the anatomical distribution of supra- and infratentorial lacunar infarcts, their demographic characteristics and acute stroke symptoms, defined by the National Institutes of Health Stroke Scale (NIHSS) score, were correlated and compared.

**Results:**

Maps of lesion distribution from 224 lacunar infarct patients (76 [33.9%] females, mean age [standard deviation] of 63.4 [11.5] years) were generated using computational image mapping methods. Median infarct volume was 0.73 ml (interquartile range [IQR] 0.37–1.15 ml). Median NIHSS sum score on hospital arrival was 4 (IQR 3–6). 165 (73.7%) patients had lacunar infarcts in the supratentorial deep white or grey matter, while 59 (26.3%) patients had infratentorial lacunar infarcts. Patients with supratentorial lacunar infarcts presented with a significantly lower occurrence of deficits in the NIHSS items gaze (*p* < 0.001) and dysarthria (*p* = 0.008), but had more often a paresis of the left arm (*p* = 0.009) and left leg (*p* = 0.068) compared to patients with infratentorial infarcts.

**Conclusions:**

The anatomical lesion distribution of lacunar infarcts reveals a distinct pattern and supports an association of localization with different stroke symptoms.

**Trial registration:**

NCT01525290.

## Introduction

Lacunar stroke, defined as small subcortical ischemic infarcts, occurs in 20 to 30% of all ischemic strokes [1]. Occlusions of perforating arteries in the deep white matter, basal ganglia, thalamus or brainstem are considered the pathophysiological mechanism [2]. Patients with lacunar infarcts often present with severe clinical deficits and benefit from systemic thrombolysis [3].

Usually determined by their clinical presentation as lacunar syndromes [4], lacunar infarcts can be verified with high sensitivity by diffusion-weighted imaging (DWI) on acute stroke magnetic resonance imaging (MRI) [5]. Applying the recently proposed imaging criteria for lacunar infarcts in the STandards for ReportIng Vascular changes on nEuroimaging (STRIVE) position paper [6], the anatomical distribution of lacunar infarcts has been previously studied by MRI with a particular focus on supratentorial locations [7–9]. Clinical characteristics and anatomical distribution patterns of infratentorial lacunar infarcts have, however, received less attention, partly due to the lack of data from imaging modalities sensitive to detect small infratentorial stroke.

We therefore aimed to map and compare the anatomical distribution of supra- and infratentorial lacunar lesions based on MRI data from a large, prospective, clinical trial. Thus, we selected patient data from the recent WAKE-UP trial (efficacy and safety of MRI-based thrombolysis in wake-up stroke). In addition, we characterized clinical presentation of patients with supra- and infratentorial lacunar stroke lesions using individual subitems from the National Institutes of Health Stroke Scale (NIHSS) score. We hypothesize that infra- and supratentorial lacunar stroke lesions lead to relevant clinical impairment with characteristic clinical phenotypes based on assessment by the NIHSS.

## Methods

### Study cohort

Data of individual MRI, performed within 4.5 h of symptom recognition, of all patients enrolled in the WAKE-UP trial was screened for acute lacunar infarcts. WAKE-UP was a multicenter-randomized, double blind, placebo-controlled trial to study MRI-based intravenous thrombolysis in acute stroke patients with unknown time of symptom onset (ClinicalTrials.gov identifier NCT01525290). The mandatory imaging criterion for randomization to treatment with alteplase or placebo was a mismatch between an acute ischemic infarct on DWI and no marked parenchymal hyperintensity in the corresponding brain region on fluid-attenuated inversion recovery (FLAIR) [10]. The detailed trial protocol and the primary results have been published previously [10]. Written informed consent, according to national and local regulations, was provided by patients or their legal representatives, with an exception from explicit informed consent in emergency circumstances in some countries. Ethics approval was obtained for each study site from the competent authorities and the corresponding ethics committee. Acute lacunar infarcts were identified as acute subcortical lesions in the territory of penetrating arteries, located in the deep white or grey matter of the cerebral hemispheres or brainstem and with a maximum diameter of 20 mm on axial plane on DWI, according to the neuroimaging criteria of the STRIVE position paper [6]. Diagnosis and anatomical lesion location of an acute lacunar infarct (supratentorial or infratentorial) was made by visual judgment and consensus of two independent neurologists with expertise in stroke MRI (E.B. and B.C.) who were blinded to clinical information. Baseline demographics and neurological deficits, assessed by the NIHSS score, were recorded on hospital arrival.

### Image processing

Individual DWI and FLAIR data were collected and data of insufficient quality excluded. Specifically, imaging artifacts (for example due to patient motion) in DWI and FLAIR sequences leading to erroneous registration were evaluated visually. Image data was analyzed using dedicated software developed for the WAKE-UP trial (Stroke Quantification Tool, SONIA) based on previous methods and functionalities of a stroke imaging toolbox developed in-house [11]. Individual DWI and FLAIR datasets were registered using a non-linear, rigid transformation. FLAIR datasets were registered to a standard template in MNI space using linear and non-linear registration. For segmentation of lacunar stroke lesions, maps of apparent diffusion coefficient (ADC) were calculated based on DWI datasets. Therefore, two datasets were chosen automatically with b-values of 0 s/mm^2^ and b-values of 500 s/mm^2^ ranging to 1500 s/mm^2^ according to the imaging protocol of the individual study site. Stroke lesions were segmented on ADC-maps using a semi-automated procedure with initial manual delineation drawing a generous margin and secondary automated refinement based on an ADC-threshold of 620 mm^2^/s. Lesion were binarized and registered to standard Montreal Neurological Institute (MNI) space using the previously generated transformation (linear and non-linear) matrices between FLAIR datasets and the MNI-template. A visual histogram overlay map was created to illustrate frequency and spatial distribution of lacunar lesions. Anatomical location of voxels with highest lesion frequencies were defined in standard MNI-space with coordinated referencing to a standard atlas of subcortical white matter fiber tracts (JHU ICBM-DTI-81 atlas) [12].

### Statistical analysis

Clinical characteristics were compared between patients with lacunar infarcts in the supratentorial white and grey matter and those with lacunar infarcts in the brainstem using the non-parametric Mann-Withney U-test for continuous outcomes, the Fisher exact test for categorical outcomes, and the Mantel-Haenszel Chi-square test for ordinal outcomes. Clinical variables were described by mean and standard deviation, median and interquartile ranges for continuous variables and with frequency and percentages for categorical variables. As all analyses were considered exploratory, all tests were carried out with a two-sided alpha level of 5% without correction for multiple comparisons.

The scores of the distinct NIHSS items (level of consciousness [item 1A], questions [1B], commands [1C], gaze [2], visual fields [3], facial palsy [4], left motor arm [5A], right motor arm [5B], left motor leg [6A], right motor leg [6B], ataxia [7], sensory [8], language [9], dysarthria [10] and extinction [11]) were dichotomised such that 0 scores represented one category (“symptom not present”) while scores > 0 were grouped into the other category (“symptom present”). Associations between patients with supratentorial lacunar infarcts and the independent variables sex, treatment, age, volume, NIHSS sum score and each NIHSS item were then individually evaluated via Mann-Whitney U- and Jonckheere-Terpstra tests and presented with the resulting Z statistics, *P* values and standardised Jonckheere-Terpstra statistics. Statistical analyses were performed with International Business Machines Corporation (IBM) SPSS statistics version 26.0 (IBM Corporation, Armonk, NY, USA 2019).

## Results

### Patient characteristics

Of 1085 patients enrolled in WAKE-UP and presenting with an acute ischemic lesion on DWI, 244 (22.5%) patients were identified with an imaging-defined lacunar infarct, of which 20 (8.2%) patients were excluded due to concurrent ischemic lesions other than lacunar infarcts. Of the 224 lacunar infarct patients included in the final analysis, 76 (33.9%) were females and had a mean age (standard deviation) of 63.4 (11.5) years. The most common cardiovascular risk factors in lacunar infarct patients were arterial hypertension (124 [55.4%] patients) and hypercholesterolemia (73 [32.6%] patients) followed by diabetes (41 [18.3%] patients). Atrial fibrillation was present in five (2.2%) patients. Median infarct volume was 0.73 ml (interquartile range [IQR] 0.37–1.15 ml). Median NIHSS sum score on hospital arrival was 4 (IQR 3–6).

Of 224 lacunar infarct patients, 103 patients were randomized to receive alteplase (54 [52.43%] patients) or placebo (49 [47.52%] patients) after screening with MRI while 121 patients were screening failures, as they did not meet all inclusion and/or exclusion criteria for inclusion in WAKE-UP. In patients randomized to treatment with alteplase, median modified Rankin Scale (mRS) score 90 days after stroke was 1.0 (IQR 0.0–2.0) compared to the median mRS score of 1.5 (IQR 1.0–2.0) in patients randomized to receive placebo.

### Anatomical distribution pattern of lesion distribution

**Fig. 1:**
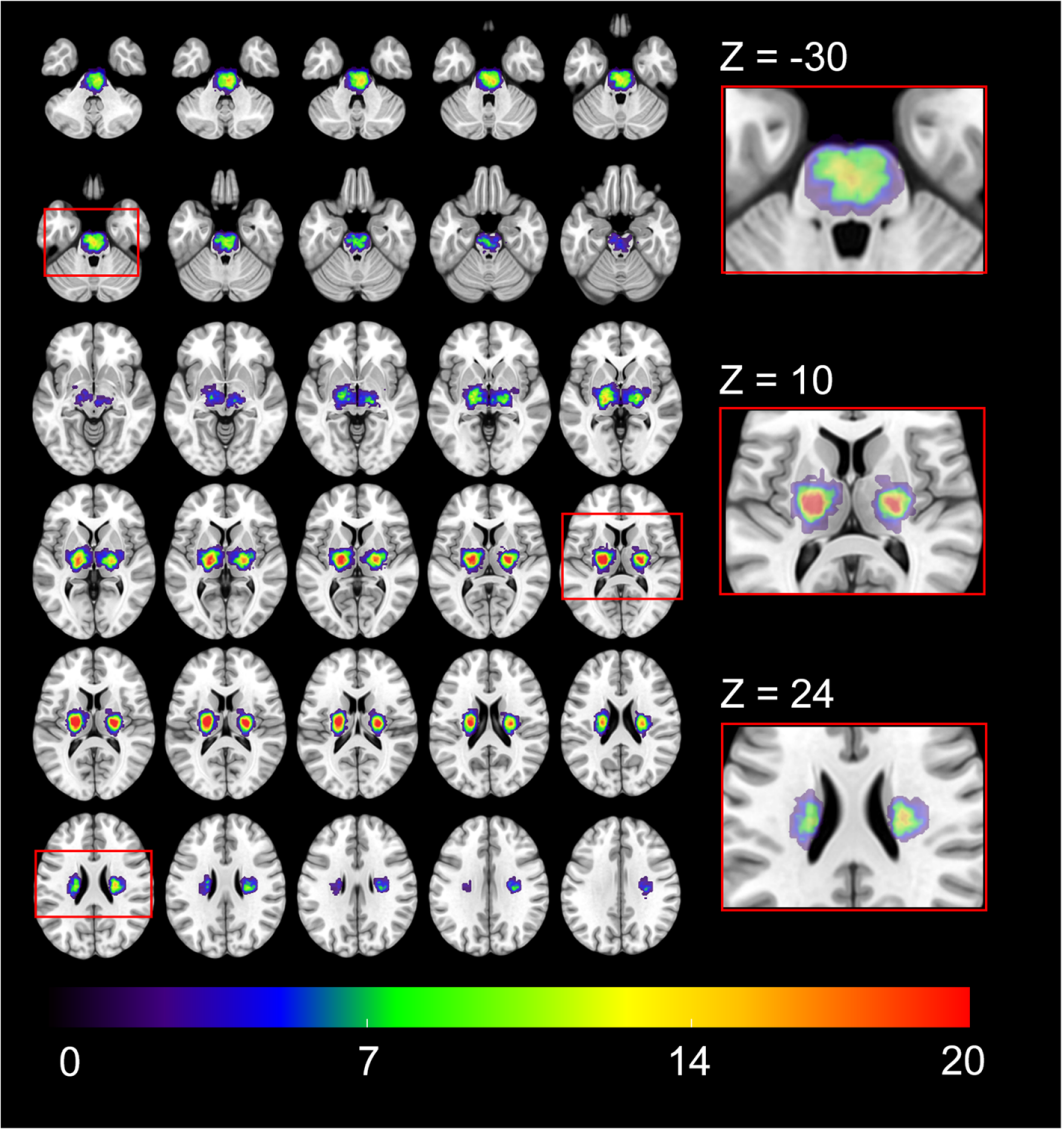
Probability distribution maps of acute lacunar infarcts. Illustration of the distribution of all lacunar infarcts in the axial plane in a standard MNI space superimposed on a mean image of spatially normalized non-diffusion-weighted (b = 0) images of all 224 patients. Z coordinates are given in millimeters

Lesion distribution was studied in all 224 patients. 165 (73.7%) lacunar infarcts were located in supratentorial deep white or grey matter or periventricular white matter with 83 (50.3%) infarcts in the left, 76 (46.1%) in the right hemisphere and six (3.6%) in both hemispheres, while 59 (26.3%) lacunar infarcts were detected in the brainstem (27 [45.8%] infarcts on the left, 29 [49.2%] on the right and three [5.1%] on both sides). Supratentorial lacunar stroke lesions occurred most commonly in the posterior limb of the internal capsule (MNI-coordinates right hemisphere: 24/-15/14; left hemisphere: − 23/-13/14) as defined by the JHU ICBM-DTI-81 white matter label atlas [12]. Of 59 infratentorial lacunar infarcts, 40 (67.8%) infarcts were located in the pons, 14 (23.7%) in the midbrain and five (8.5%) in the medulla oblongata. Overall, infratentorial lacunar lesions showed highest rates of occurrence in both corticospinal tracts (MNI-coordinates right brainstem: 4/-24/-36; left brainstem: − 4/-20/-29). Anatomical topography of lacunar lesion distribution is illustrated in Fig. 1.

### Stroke symptoms

**Fig. 2:**
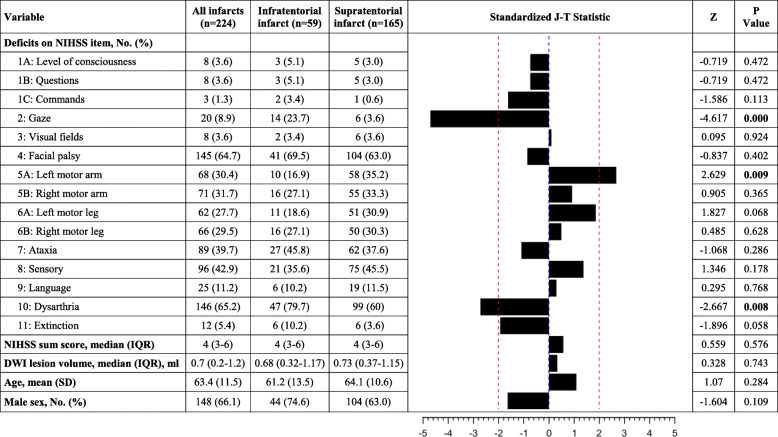
Standardized Jonckheere-Terpstra (J-T) statistics. DWI, diffusion weighted imaging; NIHSS, National Institute of Health Stroke Scale. Standardized Jonckheere-Terpstra (J-T) statistics (Z scores) depicting strength and direction of associations between supratentorially located infarcts and individual independent variables. Positive values (right section of bar graph) signify symptoms more commonly observed in supratentorial lacunar lesions. Negative values (left side of bar graph) indicate symptoms predominantly occurring in infratentorial lacunar lesions. The broken red lines mark the levels where *P* = 0.05

Comparisons of demographic data between patients with supratentorial and infratentorial lacunar infarcts revealed comparable clinical characteristics (age, gender, lesion side and lesion volume on DWI). Median NIHSS sum score was in both, patients with supratentorial and infratentorial lacunar lesions, 4 (IQR 3–6). Clinical characteristics are presented in Table 1. Patients with supratentorial lacunar infarcts presented with a significantly lower occurrence of deficits in the NIHSS item “gaze” (six patients [3.6%], *p* < 0.001) and “dysarthria” (99 patients [60%], *p* = 0.008), whereas “paresis of the left arm” (58 patients [35.2%], *p* = 0.009) and "paresis of the left leg" (51 patients [30.9%], *p* = 0.068) occurred more often as compared to patients with infratentorial lacunar infarcts. The resulting Z statistics, *P* values and standardised Jonckheere-Terpstra statistics are shown in Fig. 2.

**Table 1:** Baseline characteristics

Variable	Supratentorial infarcts (*n* = 165)	Infratentorial infarcts (*n* = 59)	*P* Value
Age, mean (SD), y	64.1 (10.6)	61.2 (13.5)	0.286
Male sex, No. (%)	104 (63.0)	44 (74.6)	0.113
Treatment allocated, No. (%)			
Alteplase	36 (21.8)	18 (30.5)	0.003
Placebo	29 (17.6)	20 (34.0)	
Not randomized	100 (60.6)	21 (35.6)	
Lesion side			
Left sided lesions, No. (%)	83 (50.3)	27 (45.8)	0.744
Right sided lesions, No. (%)	76 (46.1)	29 (49.2)	
Both sided lesions, No. (%)	6 (3.6)	3 (5.1)	
DWI lesion volume at baseline, median (IQR), ml	0.73 (0.37–1.15)	0.68 (0.32–1.17)	0.807
NIHSS score, median (IQR)	4 (3–6)	4 (3–6)	0.578

## Discussion

This post-hoc analysis of the WAKE-UP trial provides an overview of the anatomical lesion distribution pattern of supra- and infratentorial lacunar infarcts and their association with acute stroke symptoms. Our main findings demonstrate that stroke severity as measured by the NIHSS, does not differ between patients with supra- or infratentorial lacunar infarcts. There are, however some clinical characteristics specific to supra- and infratentorial infarctions: Supratentorial lacunar infarcts were more often associated with left upper extremity paresis, while infratentorial lacunar infarcts more often presented with a disturbed gaze and dysarthria. Our results, therefore, exceed the previous studies, as we not only have studied a large number of patients with infratentorial lacunar infarcts, but also the association between anatomical lesion distribution and acute stroke symptoms based on acute stroke MRI.

Lacunar infarcts have traditionally been categorized on the basis of their clinical presentation as specific lacunar syndromes. Classical lacunar syndromes comprise pure motor stroke, pure sensory stroke, sensorimotor stroke, dysarthria-clumsy-hand syndrome and ataxic hemiparesis [4]. Atypical lacunar syndromes comprise lateralized movement disorders, speech disorders and various other variations and paucisyptomatic forms of classical lacunar syndromes [13]. Although some of these syndromes are specifically attributed to supra- or infratentorial anatomical locations, there are known exceptions and variations that indicate a significant heterogeneity in lesion-symptom association. For example, oculomotor disorders are predominantly present after stroke with lesions located in infratentorial brain structures such as the pontine tegmentum [14–16], but are also reported as resulting from lacunes located in the thalamus [17, 18]. Dysarthria is mainly associated with typical lacunar syndromes located in supratentorial subcortical brain areas, but can be a symptom of an atypical lacunar syndrome with infratentorial lesion locations such as the pons and cerebellar peduncle [17–21]. Finally, motor paresis due to lacunar infarcts has been described as monoparesis or combined with dysarthria in typical lacunar syndromes, such as dysarthria-clumsy hand syndrome, usually located in the supratentorial deep white matter, but also in infratentorial stroke lesions [4, 19, 22, 23].

In line with these observations, clinical deficits caused by supra- and infratentorial lacunar stroke lesions in our group of patients showed a considerable heterogeneity. As shown in Fig. 2, distribution of specific clinical deficits in most NIHSS items was not significantly different between supra- and infratentorial lesions. There were, however, patterns that indicate some specificity of clinical symptoms regarding lesion location. There was a tendency of increase occurrence of motor symptoms, specifically left upper extremity paresis in supratentorial lacunes. Gaze disturbance, dysarthria and monoparesis in particular were associated either with supratentorial (gaze and dysarthria) or infratentorial (monoparesis) lacunar infarcts.

Although some localization of lacunar infarcts can be associated with specific clinical symptoms, misclassification of lacunar syndromes is common when compared to neuroimaging findings (clinical-imaging dissociation) [24]. Thus, a lacunar syndrome has limited specificity for the final diagnosis of a lacunar infarct [25]. The main advantage of our study is the use of MRI to diagnose acute lacunar infarctions in a large cohort, comprising the largest published cohort of acute infratentorial lacunar infarcts. Our results demonstrate that despite some specific differences in clinical symptom distribution, an overall large overlap in symptoms of lacunar stroke can be found across different lesion locations. Thus, our analysis underlines the importance of brain imaging, in particular MRI, to diagnose lacunar infarcts with a high sensitivity, to provide helpful information especially in atypical clinical presentations [5, 6, 24].

There are limitations to our study. As only patients were included with a stroke severity considerable for thrombolysis a sample bias cannot be excluded. Another sample bias might result from the patients’ age. With a mean (standard deviation) of 63.4 (11.5) years the patients assessed here were younger compared to previously described cohorts and therefore might not be completely representative for lacunar stroke patients in total. Although the NIHSS captures clinical symptoms most relevant for acute stroke treatment, we are unable to report more subtle clinical deficits such as nystagmus or detailed functional testing of cranial nerve functions. Due to the character of a post-hoc secondary analysis, no causality can be assumed from the observed association of the lesion location and neurological deficits. Our analyses were not adjusted for functional outcome, as information on outcome was available in randomized patients only. 

## Conclusion

In a large cohort of patients with acute lacunar stroke, the anatomical lesion distribution of lacunar infarcts reveals a distinct pattern and supports an association of localization with different stroke symptoms. There was, however, considerable heterogeneity in lesion-symptom associations regarding supra- and infratentorial lesions. Knowledge about the regional distribution of lacunar infarcts may provide further information on acute stroke symptoms and patient management.

## Data Availability

Not applicable.

## Abbreviations

ADC: Apparent diffusion coefficient
DWI: Diffusion-weighted imaging
FLAIR: Fluid-attenuated inversion recovery
MNI: Montreal Neurological Institute
MRI: Magnetic resonance imaging
NIHSS: National Institutes of Health Stroke Scale
STRIVE: STandards for ReportIng Vascular changes on nEuroimaging
